# A Case Report of Postcoital Dysphoria: A Paradoxical Melancholy

**DOI:** 10.7759/cureus.30746

**Published:** 2022-10-27

**Authors:** Nirnay Sachdeva, Vinay Suresh, Mohd Zeeshan, Balakrishnan Kamaraj, Abbas Mehdi

**Affiliations:** 1 Psychiatry, Career Institute of Medical Sciences & Hospital, Lucknow, IND; 2 General Medicine, King George's Medical University, Lucknow, IND; 3 Medicine, Career Institute of Medical Sciences & Hospital, Lucknow, IND; 4 Medicine, Madurai Medical College, Madurai, IND

**Keywords:** sexual health, sexual problems, male sexual health, mental health disorders, psychiatry and mental health, depression, dysphoria, postcoital

## Abstract

Sexual engagement usually leads to positive and satisfactory feelings under typical circumstances. However, studies conducted in recent years have revealed that some people experience feelings of depression, anxiety, agitation, or aggression following sexual activity or masturbation. This condition, known as postcoital dysphoria (PCD), is a rare psychiatric disorder that has been reported more in women than in men. We present a rare case of a 24-year-old male who suffers from PCD. This provides clinical insight for studies further attempting to investigate PCD among males.

## Introduction

Sexual health is an integral part of human well-being, including emotional, mental, physical, and social aspects of life. The excitation, plateau, orgasm, and resolution (EPOR) model is a four-stage "linear" model of physiological responses to sexual stimulation in the order of their occurrence proposed by William Masters and Virginia Johnson [1]. The resolution phase is characterized by euphoric feelings and sensations following satisfactory sexual activity. Research over the past couple of years, however, has shown that some individuals experience negative effects during the resolution phase, a phenomenon termed postcoital dysphoria (PCD) [1]. It is also called postcoital tristesse or "post-sex blues," which refers to feelings of deep sadness or agitation after consensual sex, even if the encounter was satisfactory and pleasurable. It is a paradoxical or counterintuitive occurrence where generally satisfying sexual activity is followed by unexplainable sensations of tearfulness, melancholy, or irritation [2]. Although Masters and Johnson's model intentionally focused on physiological changes, it was criticized for being too narrow in its conceptualization [1]. Helen Kaplan proposed a triphasic model in 1979 consisting of desire, excitement, and orgasm; the most significant contributions being the critical addition of "sexual desire" and recognizing the importance of psychological factors in sexual response [1].

There are several scales to measure the relevant clinimetrics such as the Postsex Experience Scale (P-SES) and the Arizona Sexual Experiences Scale (ASEX), and questions adopted from Bird et al.'s study, which offers a framework for evaluating the post-sex experience in both men and women and diagnose any underlying sexual dysfunctions [3-5]. Existing literature has reported that PCD affects women more than men [6]. A study reported that about 46.2% of their sample of 195 female students had experienced PCD symptoms at least once in their lifetime [2]. Maczkowiack and Schweitzer studied the prevalence and correlates of PCD among males, and reported that about 41% of 1208 male participants experienced PCD at some point in their lifetime [6]. A strong positive association was found between current psychological distress and PCD, accounting for 4.6% of the variance [6]. PCD was also linked to hypoactive sexual desire disorder, premature ejaculation, and delayed ejaculation. A weak association with childhood sexual trauma was seen, which contributed to 0.8% of the variance observed [6]. Postcoital dysphoria in men is a relatively unexplored entity and will be discussed in this case report. 

## Case presentation

A 24-year-old male law graduate visited the psychiatry outpatient department with complaints of low mood, generalized fatigue, irritability, and crying fits after having sexual intercourse in the last six months. These symptoms were insidious in onset, episodic in the course, and lasted about 30-90 minutes after intercourse. His condition deteriorated as the duration of his symptoms increased following subsequent events of intercourse. Due to these symptoms, he claimed that there was a deterioration in the relationship dynamics with his spouse that resulted in subjective discomfort for both. The patient, however, claimed to have no lack of desire to engage in sexual activity. 

He reported that he had similar feelings such as feeling uneasy and sad after masturbation at the age of 14-15 years; however, the symptoms were not so severe. As the frequency of sexual intercourse increased after marriage, the patient's symptoms worsened. The patient had a good relationship with his co-workers and had no occupational impairment. The patient was a non-alcoholic, non-smoker, and did not use any illicit drugs. He did not present with symptoms of psychological distress as per the Kessler Psychological Distress Scale [7]. Family history was not significant for any psychiatric disorder; however, the patient gave a history of being sexually abused by an adult female family member during his childhood years. The patient was cooperative, fully alert, and oriented. The patient's judgment and insight were intact as determined by the Mental State Examination (MSE) [8]. Rapport was established with ease and psychomotor activity was normal. His speech was soft and monotonous, pertinent, logical, and goal-directed with a normal reaction time. He was preoccupied with negative thoughts regarding his relationship and future but did not have any suicidal ideations. Other than post-coital mood changes and negative thoughts, the patient’s history was not suggestive of any underlying psychiatric disorders, depression, anxiety, or stress. His facial expressions, however, appeared to be sad. The patient's routine blood investigations, blood biochemistry tests, thyroid profile, liver function tests, and ECG readings were normal. 

The history given by the patient was suggestive of PCD. Other differential diagnoses included dysthymia, depression, premature ejaculation, erectile dysfunction, and hypoactive sexual desire disorder (HSDD). These conditions failed to fulfill the appropriate Diagnostic and Statistical Manual of Mental Disorders, fifth edition (DSM-5) criteria [9]. Sexual dysfunction was assessed by the ASEX, which revealed a score of 10, indicating no sexual dysfunction [4]. 

The patient was diagnosed with PCD based on the questions adopted from Bird et al.'s study: "Have there been any times in your life where inexplicable tearfulness or sadness following consensual sexual intercourse was a problem for you?" and "Have there been any times in the past four weeks where inexplicable tearfulness or sadness following consensual sexual intercourse was a problem for you?" [5]. These questions were used by Bird et al. to assess the prevalence of PCD over the lifetime and in the past four weeks [5]. 

The patient was prescribed tablet escitalopram 10 mg once a day and brief psychoeducation was instructed along with a follow-up after two weeks. The patient was a bit apprehensive during the initial phase of the treatment but later on, he got used to it. On follow-up, the patient reported decreased levels of anxiety post sexual activity. 

## Discussion

The case presented is of PCD in a male who complained of being depressed, irritated, and usually exhausted after episodes of sexual activity. The etiopathogenesis of PCD is not very well understood. Studies have only come up with possible theoretical explanations and associations. For instance, Maczkowiack and Schweitzer's study associated PCD with higher levels of psychological distress, sexual dysfunctions, and childhood sexual abuse [6]. The study examined the prevalence and correlates of PCD in a diverse sample of 1208 males of multi-national origin and found that 41% of males experienced PCD in their lifetime and 3-4% of those surveyed reported that they regularly dealt with PCD. The study concluded that the male experience of the resolution phase may be more complex in nature than previously understood [6]. 

In a study by Burri and Spector, 1489 British female twins were used as a sample to examine the experience of post-coital psychological symptoms (PPS) such as irritability and unmotivated sobbing after sexual activity and/or orgasm [10]. They found that 3.7% of women had recently experienced these symptoms, whereas 7.7% of women reported having them persistently. Recent and persistent PPS were found to be independently correlated with relationship satisfaction and abuse experience [10]. 

Burri and Hilpert conducted a study to understand the variety of post-coital symptoms in a sample of 223 women and 76 men [11]. Of these, 86.3% reported at least one symptom of “depressive mood,” 71.9% at least one symptom of “agitation,” and 71.2% reported “lethargy,” It was found that the most commonly reported symptoms in men were unhappiness and low energy whereas that in women were mood swings and sadness [11].

Our patient was diagnosed with PCD based on the questions adopted from Bird et al.'s study [5]. The scale has a Cronbach's alpha of .65 [5]. This questionnaire provides a simple, quick, and easy-to-use approach to diagnose PCD, and has been used in other studies such as the one by Schweitzer et al. [2]. 

Certain experiences such as sexual abuse during childhood can be quite impactful throughout life [12]. The patient in the current report was sexually abused by an adult family member during his adolescent years, which could have led to feelings of resentment toward sexual experiences and might have played a role in the development of PCD. This explanation is consistent with a study by Schweitzer et al., which found that a history of childhood sexual abuse seemed to be the most important predictor of PCD accounting for a 5% unique variance [2]. This finding was also seen in Bird et al.'s study where multiple regression analyses showed that a history of childhood abuse explained about 4.1% of the variance observed [5].

It has been suggested that PCD and dysfunction of the genitalia go hand in hand [5,13-14]; however, our patient had no sexual dysfunction as assessed by the ASEX. This scale has demonstrated impressive internal consistency and scale reliability, with a Cronbach’s alpha of .9055 [4]. 

Studying conditions like PCD can be quite challenging, considering the complexity and the varied number of factors that can potentially contribute to the behavioral disturbances observed in this condition, making it difficult to establish causative relations. Also, sexual health-related issues are often considered to be quite personal in nature, which can lead to a lack of clarity and thoroughness in history taking [15]. Other factors include a lack of education and the social stigma that surrounds the aspects of sexual health [15]. Healthcare providers too hesitate to initiate and explore sexual health-related problems of their patients [15]. Lack of privacy and crowded outpatient settings seem to be limiting factors as well [16]. 

The understanding of the underlying etiopathogenesis and associated risk factors remains poor due to the paucity of literature on this topic. This can undermine the quality of treatment offered to these patients; therefore, additional case reporting and future studies are necessary. Future research should adopt a biopsychosocial perspective [6,11], with a focus on PCD and partner-related variables, such as relationship quality. 

## Conclusions

PCD is an unusual condition characterized by feelings of depression and self-loathing following sexual intercourse. Patients experiencing PCD have reported mood swings, agitated behavior, and widespread exhaustion. A history of childhood sexual abuse can lead to the development of feelings of resentment toward sex and sexual experiences later in life, manifesting as postcoital dysphoria. Brief psychoeducation and selective serotonin reuptake inhibitors (SSRIs) can lead to better outcomes in such patients. Larger observational studies are required to understand the etiopathogenesis of PCD.
